# Structural characterization and antioxidant activity of pectic polysaccharides from *Veronica peregrina* L.

**DOI:** 10.3389/fnut.2023.1217862

**Published:** 2023-06-29

**Authors:** Su Yan, Xianbin Liu, Yuwen Wang, Xiaomin Yang, Lu Bai, Lin Sun, Yifa Zhou, Sisi Cui

**Affiliations:** Engineering Research Center of Glycoconjugates of Ministry of Education, Jilin Provincial Key Laboratory On Chemistry and Biology of Changbai Mountain Natural Drugs, School of Life Sciences, Northeast Normal University, Changchun, China

**Keywords:** *Veronica peregrina*, pectin, structural analysis, domain, antioxidant *Veronica peregrina*, antioxidant activity

## Abstract

**Background:**

Pectins are a class of acidic polysaccharides with complex structures. Different pectin molecules are composed of different domains, which have an important impact on their biological activity.

**Objective:**

This study aimed to determine the structural features and the antioxidant activities of the pectic polysaccharides isolated from *Veronica peregrina* L.

**Methods:**

The polysaccharide was isolated from *Veronica peregrina* L by water extraction and fractionated by ion exchange chromatography and gel permeation chromatography. The structure features of the pectic polysaccharides were determined by Fourier transforminfrared spectroscopy (FT-IR) and Nuclear magnetic resonance (NMR). The antioxidant activities was evaluated by the DPPH, OH and ABTS radical scavenging ability.

**Results:**

WVPP-A2b and WVPP-A3b, with molecular weights of 48.7 × 10^4^ and 77.6 × 10^4^  kDa, respectively, contained homogalacturonan (HG), rhamnogalacturonan I (RG-I), and rhamnogalacturonan II (RG-II) domains with a mass ratio of 2.08:2.64:1.00 and 3.87:4.65:1:00, respectively. The RG-I domain contained an arabinogalactan II backbone and arabinans consisting of t-Ara*f*, (1→5)-α-Ara*f*, and (1→3,5)-α-Ara*f*. WVPP-A3b also contained short chains consisting of the [t-Ara*f*-(1→5)-α-Ara*f*-(1→] structural unit. WVPP-A3b showed stronger ability to scavenge DPPH, hydroxyl, and ABTS radicals, which was potentially associated with its high content of galacturonic acid and presence of the HG domain.

**Conclusion:**

The results provide information for enhancing knowledge of the structureactivity relationship of pectic polysaccharides from *V. peregrina* and their potential application in the healthcare food field.

## 1. Introduction

Pectins are a class of acidic polysaccharides with complex structures that are components of plant cell walls and have diverse biological effects. Pectins primarily contain three typical domains: homogalacturonan (HG), rhamnogalacturonan I (RG-I), and rhamnogalacturonan II (RG-II). The HG domain, which accounts for ~65% of the total pectin, is a linear polysaccharide and composed of α-(1 → 4)-GalA*p*, and some galacturonic acid (GalA) residues are methylated at *O*-3 and acetylated at the *O*-2 or *O*-3 position (1). The RG-I domain (20%–35% of the total pectin) is composed of [→2)-α-Rha*p*-(1 → 4)-α-GalA*p*-(1→] repeated units as the backbone with side chains attached at C4 of (1 → 2)-α-Rha*p*. The side chains attached to RG-I are composed of arabinan, galactan, arabinogalactan-І (AG-І), and arabinogalactan-II (AG-II). The proportion of neutral sugar side chains varies from 25% to 80% depending on the source and extraction method of the pectin. The RG-II domain is a highly conserved sequence in plant cell walls and exhibits high similarity among plant species (2).

Pectins have a notable ability to scavenge free radicals because of the high GalA contents. Therefore, pectin is the focus of increasing attention as a potential antioxidant because of its unique physicochemical properties and low toxicity (3). As a biological macromolecule with a complex structure, the antioxidant activity of pectin largely depends on its structural characteristics, such as the monosaccharide composition, glycosidic bond type, sequence and configuration of sugar residues, branching degree, and substitution (4). Plant pectins have both common structural characteristics and individual characteristics peculiar to different plant sources (4). In addition, the composition and structural differences of the domains have an important impact on the biological activity of pectin. Although the main characteristics of the chemical structure of pectins have been clarified, subtle structural characteristics vary among plant species, such as the side-chain composition, esterification degree, molecular weight, branching degree, and chain conformation (5). Therefore, research on the fine structure of different plant pectins is crucial to evaluate the structure–activity relationship, and is important for the utilization of plant pectins in medicine and the food industry.

*Veronica peregrina* is an annual or biennial plant and a traditional Chinese medical herb, which belongs to the Scrophulariaceae family and is related to the widespread weed *V. polita*. *Veronica peregrina* is often used for treatment of oligomenorrhea, dysmenorrhea, and to lower blood pressure (6). To date, almost 20 compounds that exhibit antioxidant activity, such as protocatechuic acid, luteolin, veronicoside, and minecoside, have been isolated and identified from *V. peregrina* (7). However, few studies have examined the structure of pectic polysaccharides from *V. peregrina*, and especially lacking are studies of their structure–activity relationship. Therefore, in this study, pectins were extracted from *V. peregrina* and the structure was determined using a combination of chemical and instrumental methods. In addition, the antioxidative activity of *V. peregrina* pectin and its structure–activity relationship was investigated.

## 2. Materials and methods

### 2.1. Materials

Plant material of *Veronica peregrina* was purchased from Anhui province, China and stored in the herbarium of our laboratory. DEAE cellulose and Sepharose CL-6B were purchased from GE-Healthcare (United States). Monosaccharide standards were purchased from Sigma. All other chemicals were of analytical grade.

### 2.2. General methods

Total sugar content was measured using the phenol–sulfuric acid method, taking the mixture composed of the major monosaccharides as a standard (8). The uronic acid content was determined using the *m*-hydroxydiphenyl method taking GalA as the reference (9). Ultraviolet (UV) analysis was performed on a UV-2700 full wavelength UV scanner (Shimadzu, Japan). The absorbance ranging from 200 to 800 nm was recorded. The homogeneity and molecular weight (*Mw*) were determined using a Shimadzu high-performance liquid chromatography (HPLC) system equipped with a RID-20A UV detector and a TSKgel G3000PWXL column (7.8 cm × 30.0 cm). Detection of 3-deoxy-D-manno-2-octulosonic acid (Kdo) was conducted using the thiobarbituric acid (TBA) method described by York et al. (10).

### 2.3. Preparation of pectin from *Veronica peregrina*

#### 2.3.1. Extraction of pectin

The *V. peregrina* material was extracted with hot water in accordance with the protocol used in our library (5). Briefly, the dried material (1,000 g) was crushed, soaked in deionized water (16 L) and extracted at 100°C for 3 h. The process was repeated three times under the same conditions. The supernatant was collected and evaporated to 2 L at 80°C, then precipitated with 8 L of 95% ethanol, and incubated overnight at 4°C. The precipitates were washed with 95% ethanol and anhydrous ethanol in turn, and dried under vacuum at 60°C overnight to give the crude polysaccharide WVPP.

#### 2.3.2. Fractionation of the pectin

WVPP (50 g) was fractionated using a DEAE-cellulose column (12 cm × 43 cm, Cl^−^). The crude polysaccharide was eluted first with 4.5 L deionized water to obtain the neutral fraction (WVPP-N). The column was further eluted using 3 L of 0.5 M NaCl to give the crude pectin (WVPP-A).

**Figure 1:**
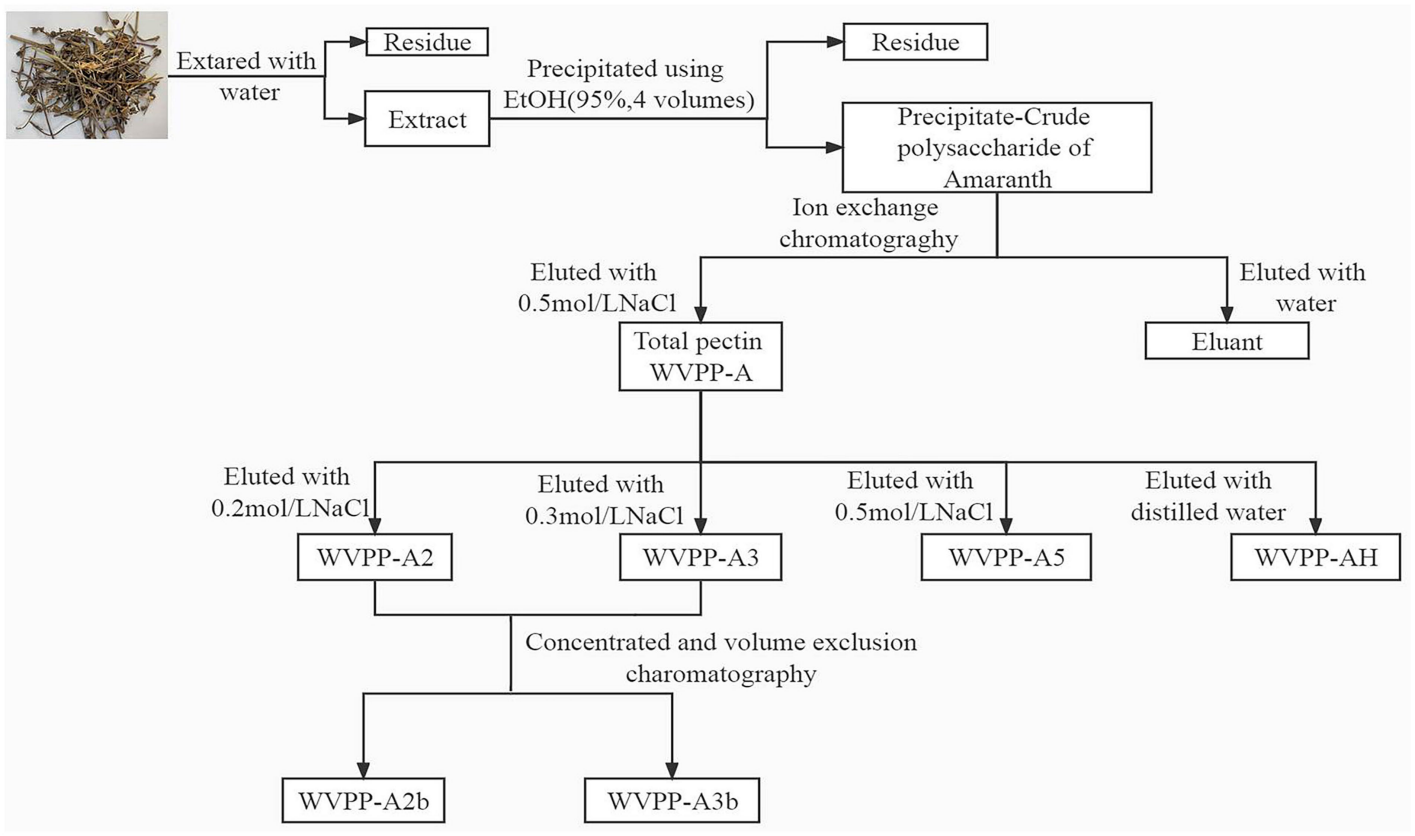
Isolation and purification of the pectic polysaccharides WVPP-A2b and WVPP-A3b from *Veronica peregrina*.

WVPP-A (1 g) was fully dissolved in distilled water, centrifuged, and loaded onto a DEAE-cellulose column (12 cm × 43 cm). The crude pectin was eluted with deionized water, then 0.2 M, 0.3 M, and 0.5 M NaCl solution in sequence. The flow rate was 25 mL/min. The total sugar content and uronic acid content in the eluate were detected, and the corresponding elution fractions were collected and designated WVPP-A2, WVPP-A3, and WVPP-A5. WVPP-A2 and WVPP-A3 were further fractionated using a Sepharose CL-6B column (3.0 cm × 100 cm) and were eluted with 0.15 M NaCl at a flow rate of 0.5 mL/min. The two purified pectic polysaccharides WVPP-A2a and WVPP-A2b were obtained. The method of extraction and fractionation of the pectins from *V. peregrina* is summarized in Figure 1.

### 2.4. Chemical characterization analysis

A polysaccharide sample (2–4 mg) was hydrolyzed with 2 M anhydrous HCl–methanol solution and trifluoroacetic acid (TFA) in accordance with a previously reported method (11). The hydrolyzed polysaccharide was added to 1-phenyl-3-methyl-5-pyrazolone (PMP) and reacted at 70°C for 30 min. The PMP derivatives were purified by chloroform extraction, then analyzed using a HPLC system equipped with a SPD-20A UV–visible diode-array detector and a COSMOSIL 5C18-PAQ column. A volume (0.1 mol/L) of PBS (pH 6.9) solution containing 17% acetonitrile (v/v) was used as the mobile phase. The column temperature, detection wavelength, and flow rate were 35°C, 245 nm, and 1 mL/min, respectively.

### 2.5. Fourier transform–infrared spectroscopy

Fully dried samples (2 mg) were mixed thoroughly with potassium bromide (1:100, w/w) and determined using a Spectrum Two FT–IR spectrometer (PE, United States) in the range of 4,000–500 cm^−1^.

### 2.6. Nuclear magnetic resonance analysis

Samples (20 mg) were dissolved in 0.5 mL D_2_O (99.9%). The ^1^H, ^13^C NMR, ^1^H-^1^H COSY, ^1^H-^13^C HSQC, and HMBC spectra were recorded with a Bruker AV600 MHz NMR spectrometer (Germany) at 25°C.

### 2.7. De-esterification and enzymatic hydrolysis

WVPP-A2b and WVPP-A3b were saponified with precooled 0.1 M NaOH and incubated at 4°C for 4 h with mild stirring (5). The reaction solutions were neutralized with 10% glacial acetic acid to pH 7.0, desalted on a Sephadex G-10 column, and freeze-dried to obtain the de-esterified pectins (WVPP-A2b-D and WVPP-A3b-D).

WVPP-A2b-D and WVPP-A3b-D were dissolved in 50 mM HAc–NaAc solution (pH 4.5). Endo-polygalacturonase M2 (EC 3.2.1.15; 50 μL) was added to the solution and incubated at 40°C for 24 h, then the reaction solution was heated in a boiling water bath for 15 min to inactivate the pectinase. The enzymatic hydrolysates were separated with a Sephadex G-75 column (2.6 cm × 100 cm) and were eluted with 0.15 M NaCl at 0.4 mL/min. The corresponding eluent was collected, desalted using a Sephadex G-10 column, and freeze-dried. Three subfractions were obtained from each of WVPP-A2b (WVPP-A2b-E1–E3) and WVPP-A3b (WVPP-A3b-E1–E3).

### 2.8. Antioxidant activity analysis

#### 2.8.1. DPPH radical-scavenging activity

The ability of the pectin fractions to scavenge the DPPH radical was determined in accordance with the method described by Chattopadhyay et al. (12). Briefly, 500 μL pectin solution at different concentrations (0.5, 1, 2, 5, and 10 mg/mL) was mixed with 2 mL of 0.5 mM DPPH solution. The resulting mixture was incubated in the dark for 30 min, then the absorbance was measured at 517 nm. Ascorbic acid (Vc) was used as a positive control, ultrapure water was used as a blank control, and an equal volume of anhydrous methanol instead of the DPPH solution served as an additional blank control. Each sample was repeated five times. The DPPH-scavenging activity of the fractions was calculated with the following formula:


$$\begin{array}{l} \mathrm{DPPH}-\mathrm{scavenging activity}\;\left(\%\right)\\ =\left[1-\left(A_{\mathrm{samples}}-A_{\mathrm{control}}\right)/A_{\mathrm{blank}}\right]\times 100\% \end{array}$$

A_samples_: The absorbance value of sample solution.
A_control_: The absorbance value of background solution (anhydrous methanol instead of the DPPH solution).
A_blank_: The absorbance value of the blank control.

#### 2.8.2. Hydroxyl radical-scavenging activity

The ability of the pectin fractions to scavenge hydroxyl (OH) radicals was assessed in accordance with a previously described method (13). The 100 μL pectin solution at different concentrations (0.5, 1, 2, 5, and 10 mg/mL) was mixed with an equal volume of FeSO_4_ solution (9.0 mmol/L) and absolute ethanol containing salicylic acid solution (9.0 mmol/L). The resulting mixture was reacted with 100 μL H_2_O_2_ solution (8.8 mmol/L) in a reaction tube at 25°C for 30 min. The absorbance at 532 nm was recorded. Ultrapure water was used as the blank control solution instead of the pectin sample, Vc was used as a positive control, and an equal volume of ultrapure water instead of the H_2_O_2_ solution served as an additional control. The hydroxyl radical-scavenging ability was calculated as follows:


$$\begin{array}{l} \mathrm{OH}-\mathrm{scavenging activity}\;\left(\%\right)\\ =\left[1-\left(A_{\mathrm{samples}}-A_{\mathrm{control}}\right)/A_{\mathrm{blank}}\right]\times 100\% \end{array}$$

A_samples_: The absorbance value of sample solution.
A_control_: The absorbance value of background solution (ultrapure water instead of the H_2_O_2_ solution).
A_blank_: The absorbance value of blank control.

#### 2.8.3. ABTS radical-scavenging activity

The ability of the pectin fractions to scavenge ABTS radicals was evaluated in accordance with a previously reported method (14). The ABTS working solution was prepared fresh daily consistent with a previously reported (14). To evaluate the ABTS radical-scavenging ability of the fractions, 400 μL of the sample solution at different concentrations (0.5, 1, 2, 5, and 10 mg/mL) was fully mixed with 400 μL ABTS working liquid in a reaction tube. The reaction was conducted in the dark for 30 min at 25°C. The absorbance at 732 nm was recorded. Equal volume of ultrapure water was used as the blank control solution instead of the samples. The ABTS radical-scavenging activity of the fractions was calculated with the following formula:


$$\begin{array}{l} \mathrm{ABTS}-\mathrm{scavenging activity}\;\left(\%\right)\\ =\left[1-\left(A_{\mathrm{samples}}-A_{\mathrm{control}}\right)/A_{\mathrm{blank}}\right]\times 100\% \end{array}$$

A_samples_: The absorbance value of sample solution.
A_control_: The absorbance value of the background solution (PBS solution instead of ABTS working solution).
A_blank_: The absorbance value of blank control.

### 2.9. Statistical analysis

The data for antioxidant effect are expressed as the mean ± SD. All assays were performed as three parallel experiments. The experimental data were analyzed using IBM SPSS Statistics 23.0 software.

## 3. Results and discussion

### 3.1. Preparation of pectic polysaccharides from *Veronica peregrina*

The crude polysaccharides (WVPP) were extracted from *V. peregrina* by hot water extraction and ethanol precipitation with a yield of 6.9% relative to the dry mass. The proportions of total carbohydrates, total protein, and glucuronic acid in the WVPP were 41.2%, 27.0%, and 5.1%, respectively (Table 1). Monosaccharide analysis revealed that the WVPP contained GalA, Ara, Glc, Gal, Rha, GlcA, Man, and Xyl in molar ratios of 25.5:17.6:17.1:13.0:8.9:8.0:7.5:2.3.

**Table 1:** Yield, molecular weight (*Mw*), and monosaccharide composition of pectic polysaccharides extracted from *Veronica peregrina.*

	WVPP	WVPP-N	WVPP-A	WVPP-A2b	WVPP-A3b
Yield (w%)	6.9^a^	49.3^b^	21.3^b^	28.1^c^	9.4^c^
Mw (kDa)		ND	ND	48.7	77.6
**Monosaccharide composition**					
GalA	25.5	-	37.6	43.7	47.4
Rha	8.0	-	15.1	13.3	18.7
Gal	17.1	26.2	15.1	16.9	11.7
Ara	13.0	11.8	16.0	15.8	12.6
Glc	17.6	53	2.0	0.9	1.7
GlcA	7.5	-	4.4	1.7	2.5
Xyl	8.9	4.8	8.7	6.7	4.1
Man	2.3	4.2	0.7	0.9	1.2

a Yield relative to the dry weight of the plant material.

b Yield relative to WVPP.

c Yield relative to WVPP-A.

**Figure 2:**
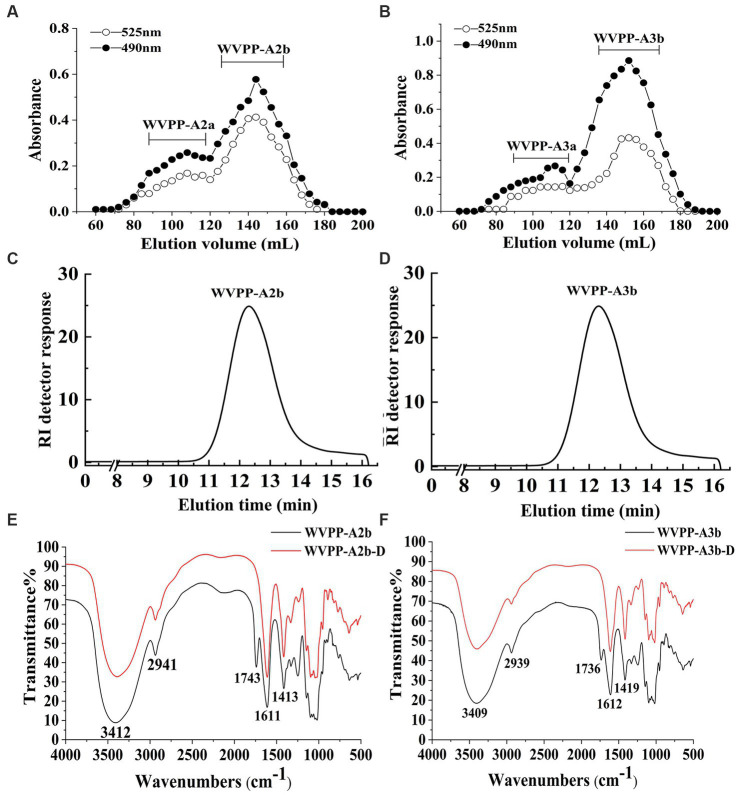
Characteristics of the WVPP-A2b and WVPP-A3b fractions. **(A,B)** Sepharose CL-6B elution curve; **(C,D)** HPGPC profiles; **(E,F)** FT-IR spectra.

The WVPP was then fractionated by ion-exchange chromatography to obtain a neutral fraction (WVPP-N) with a yield of 49.3% (relative to the WVPP) and an acidic fraction (WVPP-A) with a yield of 21.3% (relative to the WVPP). WVPP-A was further fractionated with a DEAE-cellulose column, giving WVPP-AH (8.3%), WVPP-A2 (40.0%), WVPP-A3 (12.5%), and WVPP-A5 (4.1%). Subsequently, WVPP-A2 and WVPP-A3 were further purified using a Sepharose CL-6B column (Figures 2A,B), to yield the two major fractions WVPP-A2b (70.0%) and WVPP-A3b (75.0%). The monosaccharide compositions of WVPP-A2b and WVPP-A3b were similar; both fractions were predominantly composed of GalA, Rha, Ara, and Gal (collectively comprising approximately 90% of the total monosaccharides), and small amounts of Man, Xyl, Glc, and GlcA (Table 1). The presence of GalA, Rha, Gal, and Ara may reflect the presence of HG and RG-I domains in both WVPP-A2b and WVPP-A3b. In the TBA assay, WVPP-A2b and WVPP-A3b displayed positive results, indicating that both contained a RG-II-type pectin domain.

### 3.2. Purity, homogeneity, and molecular weight of WVPP-A2b and WVPP-A3b

WVPP-A2b and WVPP-A3b showed no absorbance at 260 and 280 nm, indicating the fractions were free of nucleic acids and proteins (Supplementary Figure S1). Therefore, the purification process yielded pectin of high purity. In this study, high-performance gel permeation chromatography (HPGPC) was first used to analyze the homogeneity of WVPP-A2b and WVPP-A3b. The HPGPC elution curves of the two pectin fractions showed narrow, single symmetrical peaks (Figures 2C,D), indicating that the fractions were homogeneous with *Mw* of 48.7 × 10^4^ kDa (WVPP-A2b) and 77.6 × 10^4^ kDa (WVPP-A3b).

### 3.3. FT-IR analysis of WVPP-A2b and WVPP-A3b

As a convenient and effective method for characterization of the primary structure of polysaccharides, FT-IR can be used to detect the glycosidic bond types, sugar residue substitution, and configuration of sugar residues in polysaccharides (15). The WVPP-A2b and WVPP-A3b fractions exhibited similar FT-IR spectra (Figures 2E,F). The stronger absorption peaks at 3,412 and 3,409 cm^−1^ were attributed to the O−H stretching vibration characteristic peak of the hydrogen bond of sugar residues. The weak absorption peaks near 2,941 and 2,939 cm^−1^ were caused by the asymmetric C−H stretching vibration of the –CH_2_ groups. The absorption peaks near 1,245, 1,743, and 1,611 cm^−1^ in the spectra indicate the presence of uronic acid (16). Among these peaks, the vibration absorption peaks near 1749 cm^−1^ and 1,611 cm^−1^ represent the characteristic peaks of C=O vibration in methyl-esterified and free carboxyl groups, respectively (17). The peak areas of these two characteristic peaks can be used to calculate the degree of methylation (DM) of acidic polysaccharides. The esterification degree of pectin can be calculated with the following formula: DE (%) = [*A*_1740_/(*A*_1740_ + *A*_1611_)] × 100%, where A_1740_ is the characteristic peak area of esterified carboxyl groups (1740 cm^−1^) and A_1610_ is the characteristic peak area of free carboxyl groups (1,610 cm^−1^). Applying this formula, the DM of WVPP-A2b and WVPP-A3b was 18.7% and 19.6%, respectively.

### 3.4. NMR analysis of WVPP-A2b and WVPP-A3b

#### 3.4.1. NMR analysis of WVPP-A2b

**Figure 3:**
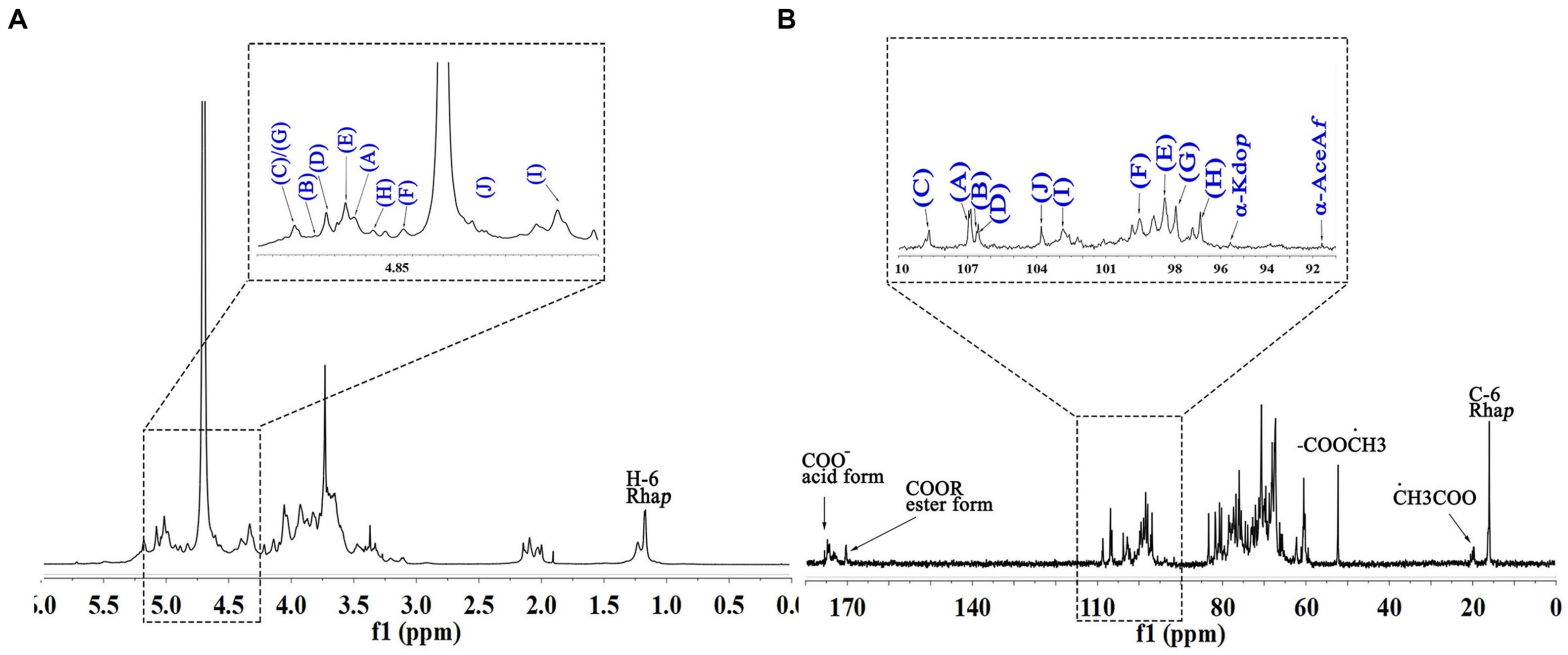
1D NMR spectra of WVPP-A2b. **(A)** ^1^H spectrum; **(B)** ^13^C spectrum.

**Figure 4:**
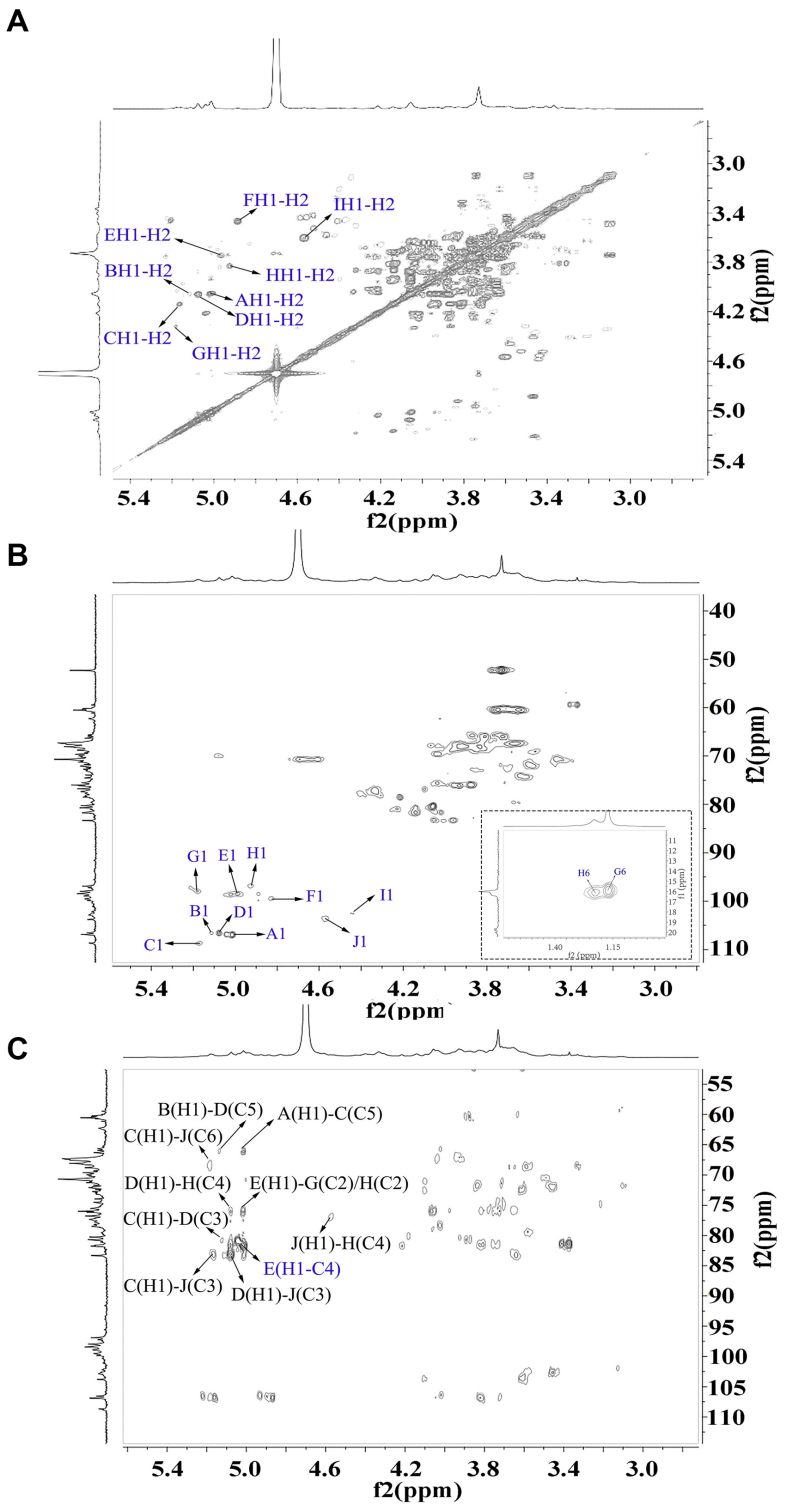
2D NMR spectra of WVPP-A2b. **(A)** ^1^H-^1^H COSY; **(B)** ^1^H-^13^C HSQC spectrum; **(C)** HMBC spectrum.

The signals at 4.57–5.17 ppm in the ^1^H spectrum (Figure 3A; Table 2) were attributed to anomeric protons, indicating the presence of α- and β-glycoside residues in WVPP-A2b (18). The signals at 19.99 and 51.95 ppm in the ^13^C spectrum were attributed to −OAc and −OCH_3_ groups (19). Small peaks observed at 95.65 and 91.97 ppm belonged to C2 of α-Kdo*p* and α-Ace*f*A (5), indicating the presence of a RG-II domain in WVPP-A2b (Figure 3B), which was consistent with the TBA detection results. Based on the ^1^H, ^13^C, ^1^H-^1^H COSY (Figure 4A), and ^1^H-^13^C HSQC (Figure 4B) spectra, as well as published data for pectins, the chemical shifts of all carbon and hydrogen atoms in the sugar residues of WVPP-A2b were assigned and are listed in Table 2. The anomeric proton and carbon signals at 5.00/106.93, 5.11/106.62, 5.17/108.64 ppm, and 5.08/106.57 ppm confirmed that the Ara*f* residues in WVPP-A2a were in the form of α-Ara*f*. The signals at 4.01/83.40, 3.88/75.96, 4.06/80.34 ppm, and 3.66/60.52 ppm were attributed to the H2/C2-H5/C5 of residue A, indicating that residue A was t-α-Ara*f*. Based on the ^1^H-^13^C HSQC spectrum, the signal at 3.72/66.01 ppm was assigned to the H5/C5 of residues C and D. In comparison with the chemical shifts of residue A, the chemical shift of C5 of residue C shifted down-field, indicating the presence of a substitution at C5 of the residue. Therefore, residue A was assigned to α-(1 → 5)-Ara*f*. Similarly, the down-field shift of C3 (83.15 ppm) and C5 (66.01 ppm) in residue D was observed, indicating that residue D was α-(1 → 5)-Ara*f* and α-(1 → 3,5)-Ara*f* (20). In the ^13^C NMR spectrum, the signals at 170.14 and 174.86 ppm were assigned to esterified and non-esterified carboxyl groups of Gal*p*A (21). The presence of signals at 5.01/98.46, 4.83/99.46, 4.02/81.63, and 4.05/80.51 ppm confirmed that residues E and F were α-(1 → 4)-Gal*p*A and α-(1 → 4)-GalA6Me*p*, respectively. The signals at 5.17 and 4.93 ppm indicated that residues G and H were α-Rha*p* (22). The cross peaks of H1/C1 for (1 → 2)-α-D-Rha*p* and (1 → 2,4)-α-D-Rha*p* were observed at 5.17/97.91 and 4.93/96.88 ppm, respectively (Figure 4B), and H6/C6 was observed at 1.17/15.89 and 1.23/16.12 ppm, respectively. The signal at 5.47/103.64 ppm was assigned to H1/C1 of residue I, indicating that it was in the β-configuration. The chemical shifts of C3 (83.15 ppm) and C6 (68.24 ppm) in residue G were down-field relative to the chemical shift of β-Gal, indicating that residue G was (1 → 3, 6)-β-D-Gal*p* (23).

**Table 2:** ^1^^3^C and ^1^H NMR chemical shift assignments of the residues in WVPP-A2b.

Residues	Glycosidic linkage		1	2	3	4	5	6
A	α-Ara*f-*(1 → ^AtІ^	H	5.00	4.01	3.88	4.06	3.66	
		C	106.93	83.40	75.96	80.34	60.52	
B	α-Ara*f-*(1 → ^AtII^	H	5.11	3.96	3.88	4.06	3.74	
		C	106.62	83.27	75.96	80.34	60.44	
C	→5)-α-Ara*f-*(1→	H	5.17	4.23	4.03	4.22	3.72	
		C	108.64	80.91	75.58	80.91	66.01	
D	→3,5)-α-Ara*f-*(1→	H	5.08	4.31	4.06	3.96	3.72	
		C	106.57	78.16	80.39	83.27	66.01	
E	→4)-α-GalA*p-*(1→	H	5.01	3.67	4.04	4.02	4.69	
		C	98.46	67.41	69.58	81.63	70.69	174.86
F	→4)-α-GalA6Me*p-*(1→	H	4.83	3.67	4.04	4.05	4.60	
		C	99.46	67.41	69.58	80.51	70.73	170.14
G	→2)-α-Rha*p-*(1→	H	5.17	4.33	4.04	3.57	3.46	1.17
		C	97.91	76.14	69.37	71.90	70.60	15.89
H	→2,4)-α-Rha*p-*(1→	H	4.93	4.40	4.02	3.93	3.57	1.23
		C	96.88	76.14	73.44	77.77	69.10	16.12
I	→3)-β-Gal*p*-(1→	H	4.42	3.58	4.05	4.02	3.61	3.52
		C	102.55	71.79	83.26	68.18	74.43	70.84
J	→3,6)-β-Galp-(1→	H	4.57	4.01	4.08	4.07	3.62	3.83
		C	103.64	73.68	83.08	67.83	74.26	66.09

As observed in the HMBC spectrum (Figure 4C), the cross peak at 5.01/81.67 ppm was attributed to EH1-EC4, indicating the presence of a HG domain. The EH1-GC2, EH1-HC2, GH1-EC4, and HH1-EC4 indicated the presence of the [→4)-Gal*p*A-(1 → 2)-Rha*p*-(1 → 4)-Gal*p*A-(1 → 2,4)-Rha*p*-(1 → 4)-Gal*p*A-(1→] structural units, which formed the backbone of the RG-1 domain. The presence of AH1-CC5, CH1-DC3, and BH1-DC5 in the HMBC spectrum indicated that WVPP-A2a contained an arabinan as the side chain. In addition, the cross peaks of DH1-IC3 and CHI-IC6 indicated the presence of an arabinogalactan (AG-II) side chain in WVPP-A2a. The presence of DH1-GC4 and IH1-GC4 confirmed that the side chains in WVPP-A2a were linked to the backbone of RG-I via (1 → 3,5)-Ara*f* (D) and (1 → 3,6)-Gal*p* (I).

#### 3.4.2. NMR analysis of WVPP-A3b

**Figure 5:**
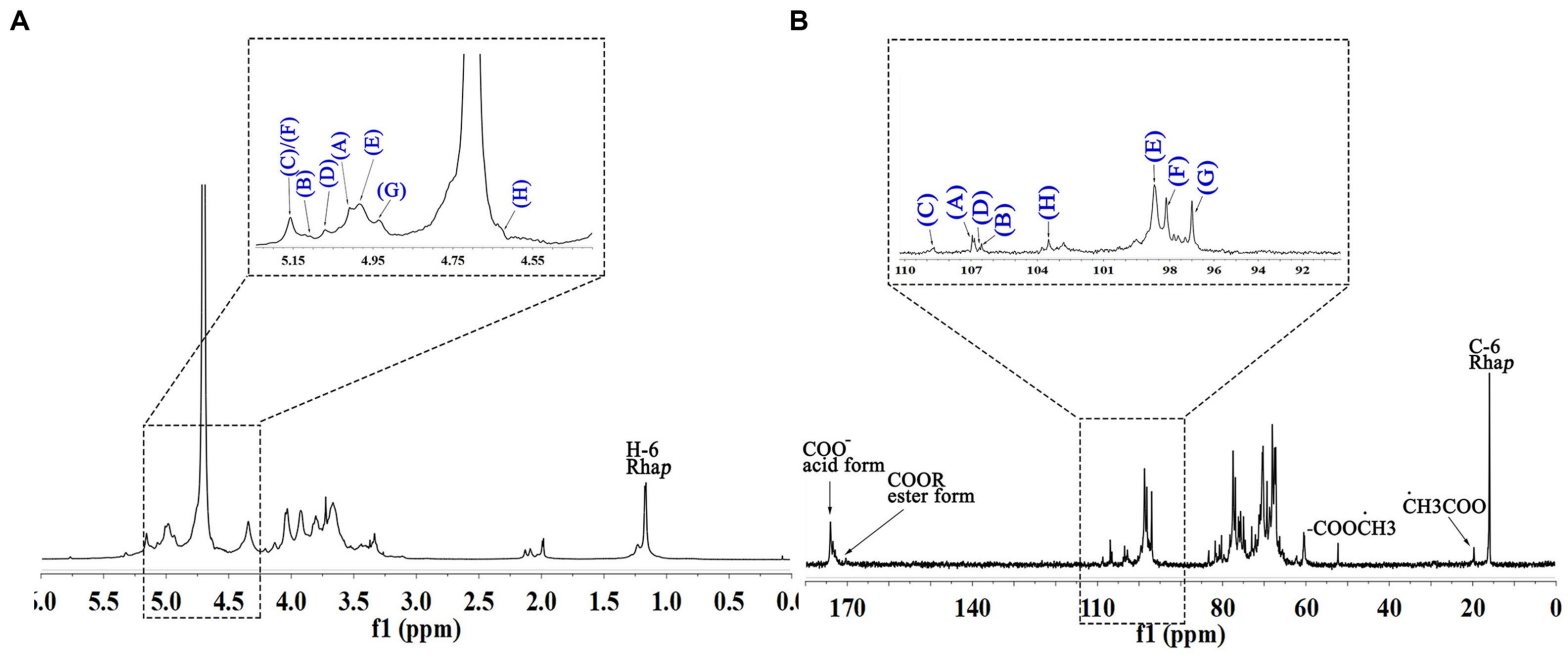
1D NMR spectra of WVPP-A3b. **(A)** ^1^H spectrum; **(B)** ^13^C spectrum.

**Figure 6:**
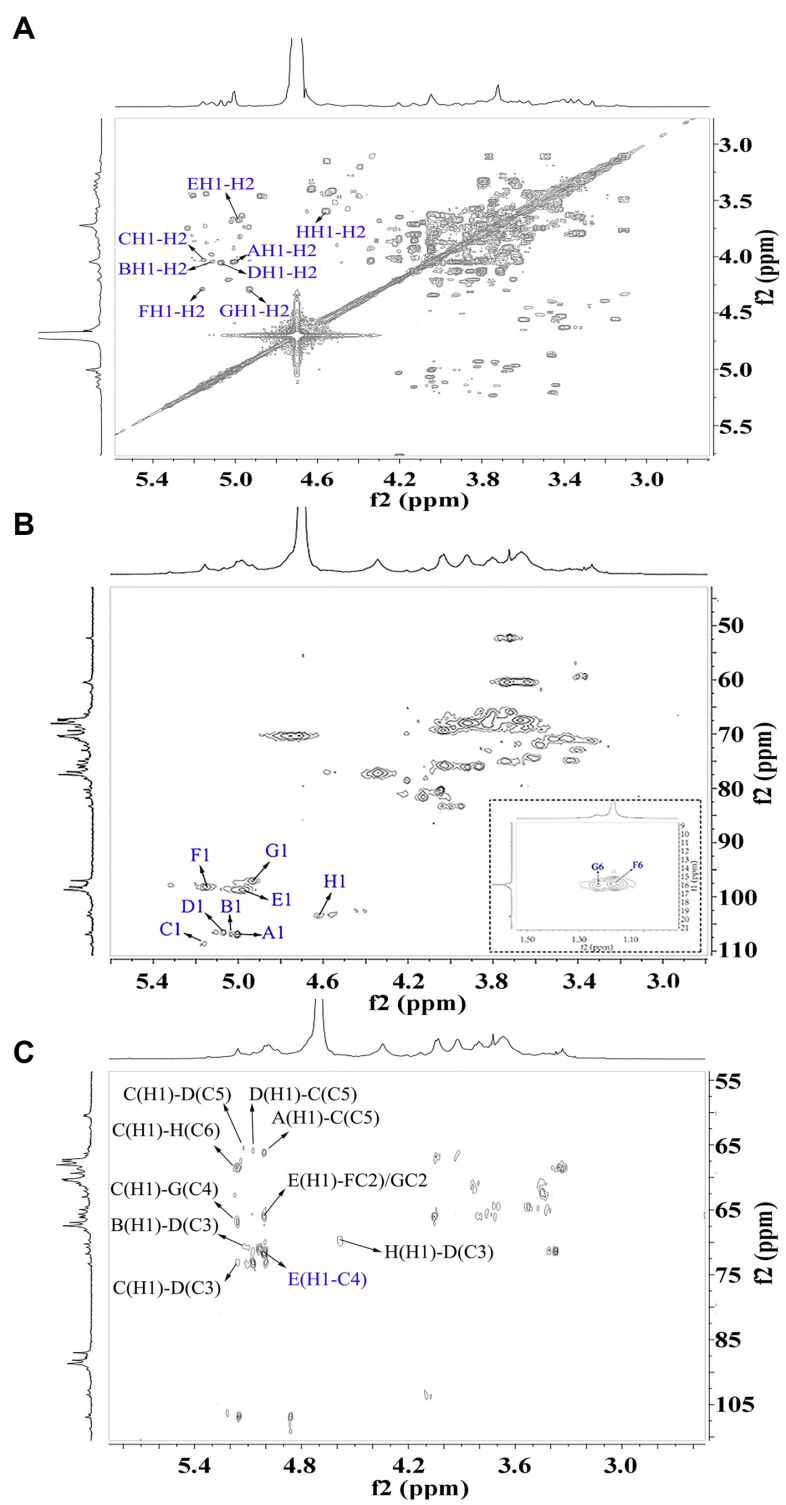
2D NMR spectra of WVPP-A3b. **(A)** ^1^H-^1^H COSY; **(B)** ^1^H-^13^C HSQC spectrum; **(C)** HMBC spectrum.

The anomeric proton and carbon regions were distributed at 95–110 ppm (Figures 5A,B; Table 3), indicating the presence of both α- and β-configurations for glycosidic linkages in WVPP-A3b. Based on the ^1^H, ^13^C, ^1^H-^1^H COSY (Figure 6A), and ^1^H-13^1^C HSQC spectra (Figure 6B), WVPP-A3b contained the same sugar residues as WVPP-A2b. The signals observed at 5.01/106.94 ppm (5.10/106.52 ppm), 5.16/108.59, 5.07/106.55, 4.98/98.69, 5.16/98.17, 4.93/97.00, and 4.9358/103.48 ppm were attributed to H1-C1 of α-t-Ara*f* (A and B), (1 → 5)-α-L-Ara*f* (C), (1 → 3,5)-α-L-Ara*f* (D), (1 → 4)-α-D-GalA*p* (E), (1 → 2)-α-D-Rha*p* (F), (1 → 2,4)-α-D-Rha*p* (G), and (1 → 3,6)-β-D-Gal*p* (H), respectively. Other chemical shifts of all residues in WVVP-A3b are listed in Table 2. Similar to WVPP-A2b, a distinct signal at 52.56 ppm was observed, indicating the presence of metylated GalA in WVPP-A3b.

**Table 3:** ^13^C and ^1^H NMR chemical shift assignments of the residues in WVPP-A3b.

Residue	Glycosidic linkage		1	2	3	4	5	6
A	α-Ara*f-*(1 → ^AtІ^	H	5.01	4.00	3.87	4.05	3.64	
		C	106.94	83.40	76.00	80.41	60.43	
B	α-Ara*f-*(1 → ^AtII^	H	5.10	4.04	3.92	4.05	3.74	
		C	106.52	83.16	76.11	80.41	60.48	
C	→5)-α-Ara*f-*(1→	H	5.16	4.22	4.03	4.22	3.72	
		C	108.59	81.10	75.70	81.59	66.14	
D	→3,5)-α-Ara*f-*(1→	H	5.07	4.21	4.06	3.95	3.72	
		C	106.55	78.51	80.60	83.28	65.53	
E	→4)-α-GalA*p-*(1→	H	4.98	3.67	4.04	4.05	4.74	
		C	98.69	67.44	69.32	81.63	70.37	174.01
F	→2)-α-Rha*p-*(1→	H	5.16	4.34	4.04	3.57	3.45	1.16
		C	98.17	76.02	69.32	72.04	70.91	15.84
G	→2,4)-α-Rha*p-*(1→	H	4.93	4.34	4.04	3.92	3.57	1.22
		C	97.00	76.02	69.32	76.85	72.04	16.05
H	→3,6)-β-Gal*p-*(1→	H	4.58	4.01	4.04	4.06	3.61	3.80
		C	103.48	75.70	83.16	67.95	74.47	68.37

The sequence of sugar residues in WVPP-A3b was analyzed based on the HMBC spectrum (Figure 6C). The cross peak at 4.98/81.63 ppm, assigned to EHI-EC4, confirmed the presence of [→4)-α-D-GalA*p-*(1 → 4)-α-D-GalA*p-*(1→] repeating units. The EH1-GC2, EH1-HC2, FH1-EC4, and EH1-EC4 indicated that (1 → 4)-α-D-GalA*p*, (1 → 2)-α-D-Rha*p*, and (1 → 2,4)-α-D-Rha*p* were linked alternately to form the backbone of the RG-I domain in WVPP-A3b. The cross peaks at 5.16/76.02 ppm (CH1-EC4) and 5.01/66.14 ppm (AH1-CC5) indicated the presence of [t-α-L-Ara*f-*(1 → 5)-α-L-Ara*f-*(1→] units connected to the main chain of RG-I via C4 of (1 → 2,4)-α-D-Rha*p*. These results showed that both pectins contained short side chains composed of α-L-Ara, which exhibited differences in the number and type of sugar residues and in the connection with the RG-I backbone. The signals at HH1-DC3 and DH1-HC3 suggested that WVPP-A2b also had AG-II side chains that contained repeating units formed by alternating connection of (1 → 3,6)-β-D-Gal*p* and (1 → 3,5)-α-L-Ara*f*, respectively, through (1 → 3)-glycosidic linkages. This result indicated that the AG-II side chains of WVPP-A3b might exhibit a higher degree of branching than those of WVPP-A2b. The signal at DH1-CC5 suggested that the AG-II side chains were connected to the backbone through (1 → 5)-α-L-Ara*f*. According to the 1D and 2D NMR results, both WVPP-A2b and WVPP-A3b contained typical HG domains, which consisted of a linear skeleton composed of [→ 4)-GalA*p*-(1 → 4)-GalA*p* (1→] structural units, with methylation and acetylation of Gal*p*A in the HG domain to different degrees. In addition, the pectins contained a similar backbone to the RG-I domain, which was composed of [→4)-α-GalA*p*-(1 → 2)-α-Rha*p*-(1→] and [→4)-α-GalAp-(1 → 2,4)-α-Rha*p*-(1→] repeating units with the branches located at C4 of α-Rha*p*. However, the branching points of RG-I differ in pectins from different plant sources. Naran et al. isolated a RG-I pectin from flax seed mucilage in which the branching point was located at C-3 of (1 → 2)-α-Rha*p* (24). Although WVPP-A2b and WVPP-A3b both contained typical HG and RG-1 domains, the neutral side chains differed and the AG-II structure in WVPP-A3b was more complex.

### 3.5. Enzymatic analysis of WVPP-A2b and WVPP-A3b

Based on the monosaccharide composition and NMR results, WVPP-A2b and WVPP-A3b both contained HG, RG-I, and RG-II domains. For further analysis of the pectin structure and function, WVPP-A2b and WVPP-A3b were hydrolyzed using endo-PG hydrolysis and the different domains were separated by HPGPC.

#### 3.5.1. Preparation of de-esterified pectin

**Figure 7:**
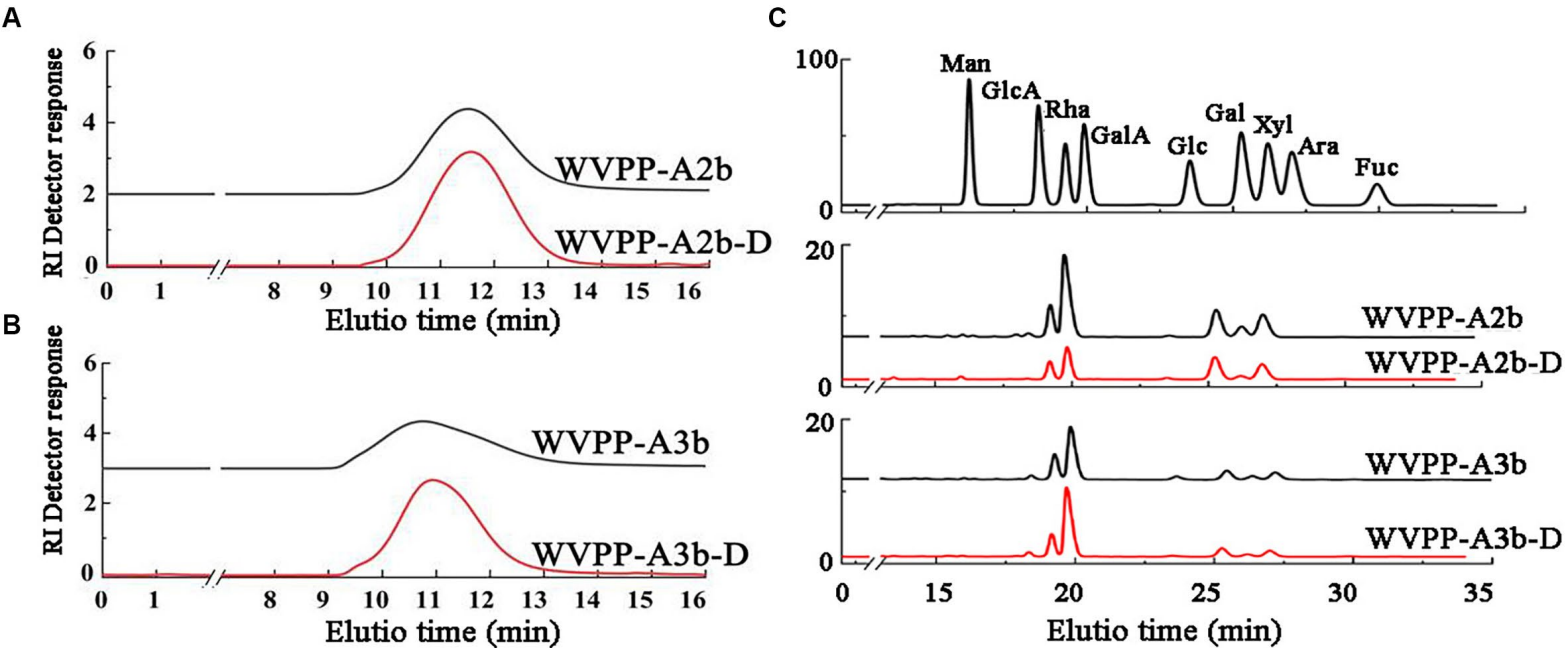
Characteristics of the de-esterified products of WVPP-A2b-D and WVPP-A3b-D. **(A,B)** HPGPC profiles; **(C)** monosaccharide composition.

Endo-polygalactonase M2 (EC 3.2.1.15) specifically recognizes and hydrolyzes the unesterified GalA in a HG-type domain. The presence of methyl and acetyl groups in HG often affects the enzymatic hydrolysis of pectinase, thereby affecting the purity of the HG domain (25). Therefore, in this study, the de-esterification of the pectin was conducted at a lower temperature in a weakly alkaline environment. FT-IR, HPGPC, and monosaccharide composition analysis were used to evaluate the de-esterification effect and the structural integrity of WVPP-A2b and WVPP-A3b. Based on the HPGPC elution profiles (Figures 7A,B), no significant change in the *Mw* distribution of WVPP-A2b and WVPP-A3b was observed after saponification treatment, suggesting that the long chain of the pectin was not broken during the saponification reaction. In addition, the monosaccharide compositions of WVPP-A2b-D and WVPP-A3b-D were not significantly changed from that of the original pectin fraction (Figure 7C). In the FT-IR spectra of WVPP-A2b-D and WVPP-A3b-D (Figures 2E,F), the signal near 1749 cm^−1^ attributed to C=O of the methylated −COO^−^ disappeared, whereas the signal near 1,610 cm^−1^ increased significantly, indicating that the methyl groups of WVPP-A2b and WVPP-A3b were removed completely.

#### 3.5.2. Analysis of enzymatic hydrolysates

Endo-PG specifically degrades unesterified GalA, degrading the HG domain to oligogalacturonide structural units, while releasing the RG-I and RG-II-type domains from the pectin. In this study, endo-PG was used to degrade the two pectins and resulted in two enzymatic hydrolysates, namely, WVPP-A2b-DE and WVPP-A3b-DE. Based on the HPGPC results (Supplementary Figure S2), the *Mw* of WVPP-A2b and WVPP-A3b changed significantly, and multiple chromatographic peaks were observed in the HPGPC profiles. WVPP-A2b-DE and WVPP-A3b-DE were further fractionated using a Sephadex G-75 column and three types of hydrolysates (E1–E3) were prepared for both WVPP-A2b and WVPP-A3b (Supplementary Figure S3).

The *Mw* of WVPP-A2b-DE1 and WVPP-A3b-DE1 was 59.7 and 73.6 kDa, respectively (Table 4). The de-esterified hydrolysates were mainly composed of GalA, Rha, Gal, and Ara, and the molar ratio of Rha/GalA was close to 1, indicating that the hydrolysates were RG-I-type pectins. WVPP-A2b-DE1 contained higher proportions of Gal (29.0%) and Ara (29.3%) than WVPP-A3b-DE1. The ratio of (Ara + Gal)/Rha reflects the average length and relative monosaccharide proportions of the neutral side chains in the RG-I domain (25). The molar ratio of (Gal + Ara)/Rha in WVPP-A2b-DE1 was 3.7, which was approximately 3.4 times higher than that of WVPP-A3b-DE1. This result indicated that the neutral sugar side chains in WVPP-A2b-DE1 were longer or more highly branched than in WVPP-A3b-DE1. Both WVPP-A2b-DE2 and WVPP-DE2 showed positive results in TBA reactions, suggesting that both are RG-II-type pectins. Double peaks were observed in the HPGPC spectra, with *Mw* distributions of 5.4–10.4 kDa. The *Mw* of WVPP-A2b-DE3 and WVPP-A3b-DE3 was less than 2.0 kDa and both hydrolysates were mainly composed of GalA (82.9%–95.9%), indicating that they were oligogalacturonides produced by endo-PG hydrolysis of the HG-type domains.

**Table 4:** Yield, molecular weight (*Mw*), and monosaccharide composition of enzymatic hydrolysates (E1–E3 fractions) of the pectins WVPP-A2b and WVPP-A3b.

Fractions	Yield^a^(w%)	TBA test	Mw (kDa)	Monosaccahride composition (mol%)							
				GalA	Rha	Gal	Ara	Glc	GlcA	Man	Xyl
WVPP-A2b-E1	46.2	−	59.7	15.8	15.6	29.0	29.3	1.5	1.0	7.8	*−*
WVPP-A2b-E2	17.5	+	9.7 and 5.4	39.1	19.8	11.9	16.7	1.9	4.0	0.8	5.8
WVPP-A2b-E3	36.4	−	<2.0	91.3	−	1.2	0.6	1.3	0.7	4.2	0.7
WVPP-A3b-E1	50.2	−	73.6	31.0	30.6	19.3	14.3	1.9	1.8	1.1	0.9
WVPP-A3b-E2	10.8	+	10.4 and 6.4	27.4	16.0	20.5	23.6	3.4	6.3	1	1.8
WVPP-A3b-E3	41.8	−	<2.0	95.9	−	0.5	0.5	1.0	0.3	1.4	0.4

a Yield relative to WVPP-A2b or WVPP-A3b.

### 3.6. Antioxidant activity analysis

**Figure 8:**
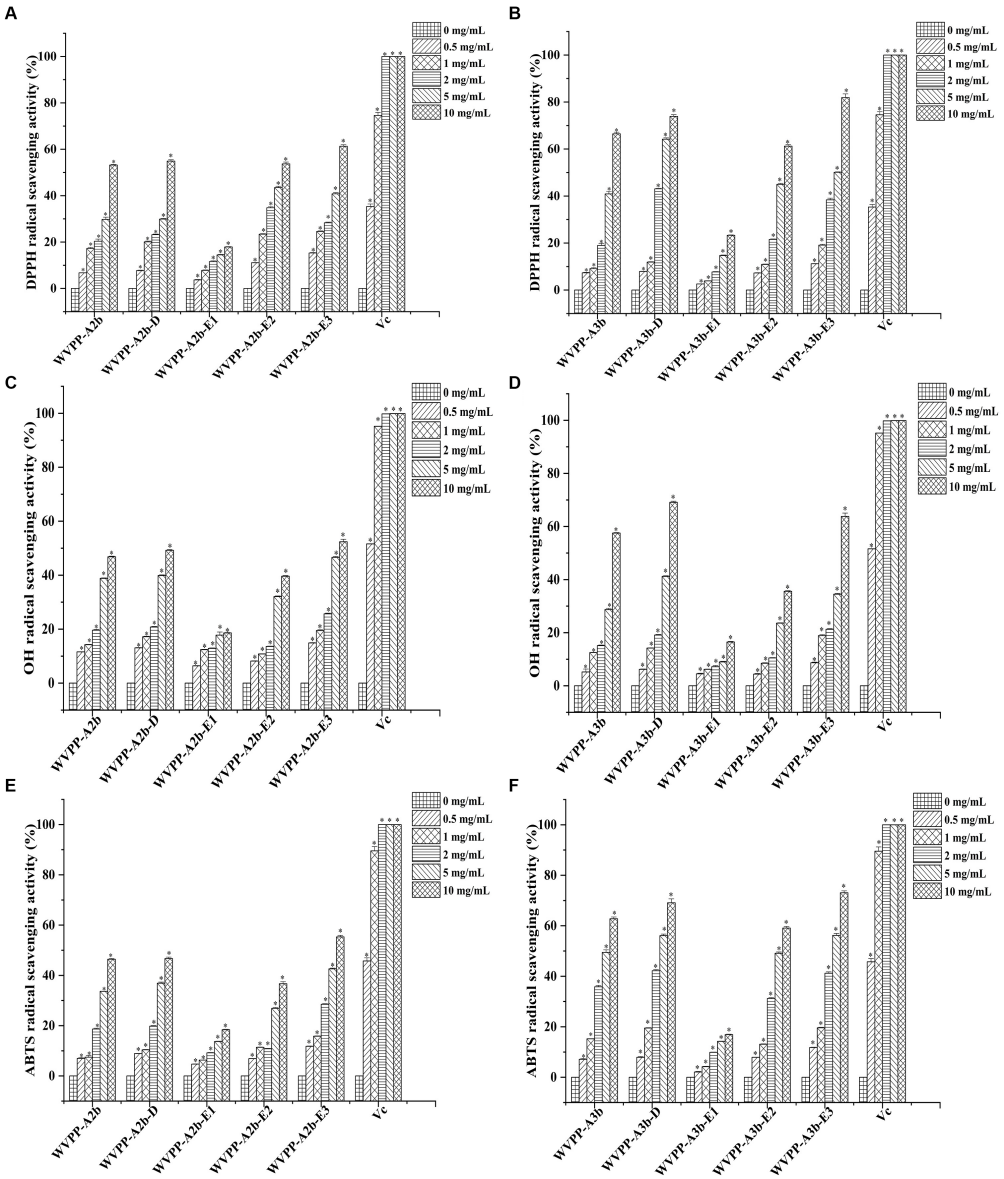
Ability of *Veronica peregrina* pectin fractions to scavenge **(A,B)** DPPH radicals, **(C,D)** hydroxyl radicals, and **(E,F)** ABTS radicals. Ascorbic acid (Vc) was used as a positive control. Each value represents the mean ± SD (*n* = 3; *p < 0.05). All experiments were performed in triplicate.

The *in vitro* antioxidant activities of WVPP-A2b and WVPP-A3b were measured as the ability to scavenge DPPH, hydroxyl, and ABTS radicals. Within the tested range (0.5–10 mg/mL), WVPP-A2b and WVPP-A3b showed significant abilities to scavenge DPPH, hydroxyl, and ABTS radicals in a dose-dependent manner (Figures 8A–F). The 50% inhibition concentrations (IC_50_) of WVPP-A2b toward the three radicals were 10.23, 11.21, and 11.33 mg/mL, respectively, whereas the corresponding IC_50_ values of WVPP-A3b were 6.22, 8.76, and 5.12 mg/mL. These results indicated that the radical-scavenging ability of WVPP-A3b was superior to that of WVPP-A2b in the present test system, but was lower than that of Vc.

To further investigate the relationship between the antioxidant activity and structure of WVPP-A2b and WVPP-A3b, the radical-scavenging abilities of the de-esterified pectins (WVPP-A2b-D and WVPP-A3b-D) and their different domains, comprising WVPP-A2b-E1 (RG-I), WVPP-A2b-E2 (RG-II), and WVPP-A2b-E3 (oligogalacturonides), were measured. The degree of methyl-esterification is an important factor affecting the antioxidant activity of pectin. Generally, the degree of methylation of the pectin plays an important role in the antioxidant activity. Wikiera et al. observed that the antioxidant activity of apple pectins was negatively correlated with the degree of methylation (26). In this study, WVPP-A2b and WVPP-A3b showed similar degrees of methyl-esterification, but WVPP-A3b showed a stronger ability to scavenge DPPH, hydroxyl, and ABTS radicals. Interestingly, after de-esterification, the antioxidant effect of WVPP-A3b was significantly enhanced; however, the effect of de-esterification on the antioxidant ability of WVPP-A2b was extremely weak. These results indicated that the relationship between the structure of the pectin and its antioxidant activity is quite complex and may be a result of the interaction of many factors. Molecular weight is an important factor affecting the antioxidant activity of polysaccharides. It is generally believed that high-molecular-weight pectin facilitates the formation of a large number of intermolecular or intramolecular hydrogen bonds, leading to a decrease in the activity of hydroxyl groups. However, pectin with a lower molecular weight may have a relatively loose conformation that facilitates the exposure of free hydroxyl groups and the reaction of radicals to remove them (27). In contrast, the monosaccharide composition of the polysaccharides plays an important role in their antioxidant activity. Li et al. found that GlcA and GalA had significant effect on the scavenging ability of *Cissus pteroclada Hayata* (CPHP) on DPPH, superoxide radical, hydroxyl radical, and ABTS radical (3). Some neutral monosaccharide, such as Gal, Ara, and Glc had significantly effects on the DPPH radical scavenging ability of polysaccharides (28, 29). The research of Qu et al. shown that 3-O-methylated-α-D-galactopyranosyl (3-O-Me-Gal*p*) in *Pleurotus Ostreatus* polysaccahride functions as an antioxidant (30). Pectins that contain a certain amount of GalA are potent antioxidants; the content of uronic acid and its degree of polymerization may impart the antioxidant activity to the pectin (31). The ability of the three domains obtained by enzymatic hydrolysis of WVPP-A2b and WVPP-A3b to scavenge free radicals is summarized in Figure 8. Within the dose range of 0.5–10 mg/mL, the radical-scavenging ability of the three domains varied greatly. The relative radical-scavenging ability was as follows: that of oligogalacturonides (which had the highest amount of GalA and the lowest *Mw*) was higher than that of the RG-II domain (E2), and the RG-I domain (E1; which had a lower content of GalA and higher number of branches) showed the lowest radical-scavenging ability. Furthermore, the radical-scavenging ability of oligogalacturonides was dose-dependent and was superior to that of the pectins (WVPP-A2b and WVPP-A3b) at the same concentration. The results were in accordance with the results of previous study, which confirmed that pectin containing higher content of GalA and HG type domains has stronger radical scavenging capacity (5, 32).

Based on these results, WVPP-A3b had stronger antioxidant activity than did WVPP-A2b, which may be due to the higher GalA content and lower molecular weight of WVPP-A3b. However, the presence of methyl groups weakened its ability to scavenge free radicals. The *in vitro* antioxidant ability of WVPP-A2b and WVPP-A3b was the result of the synergistic effects of different pectin domains, of which the HG domain contributed the most, followed by the RG-II domain; the RG-I domain, which had a higher number of branches and higher *Mw*, contributed the least to the antioxidant activity of the pectin.

## 4. Conclusion

In this study, two pectins, designated WVPP-A2b and WVPP-A3b, were prepared from *V. peregrina*, and their structural properties and antioxidant activity were investigated. The *Mw* distributions of WVPP-A2b and WVPP-A3b are 48.7 × 10^4^ and 77.6 × 10^4^ kDa, respectively. The principal monosaccharides in both pectins are GalA, Rha, Gal, and Ara, with the total proportion exceeding 80%. Both pectins contain HG, RG-I, and RG-II domains with mass ratios of 2.08:2.64:1.00 and 3.87:4.65:1:00, respectively. The branches of both polysaccharides are located at C4 of α-Rha*p*. The RG-I domains of both WVPP-A2b and WVPP-A3b contain arabinan and AG-II structures. In addition, WVPP-A2b also contains a disaccharide short side chain formed by the [t-α-Ara*f*-(1 → 5)-α-Ara*f-*(1→] structural unit. The two pectins exhibit similar methyl esterification but differ in their radical-scavenging ability. WVPP-A3b, which has a higher content of GalA and HG domain, exhibits stronger radical-scavenging activity and is a potential natural antioxidant agent for use in medicine or functional foods.

## Data availability statement

The original contributions presented in the study are included in the article/Supplementary material, further inquiries can be directed to the corresponding author.

## Author contributions

SY and XL: investigation and writing—original draft. YW and LB: investigation. YZ: formal analysis. LS and SC: writing—review and editing. All authors contributed to the article and approved the submitted version.

## Funding

This work was supported by the National Natural Science Foundation of China (no: 32271339 and 32000907) and the Scientific and Technologic Foundation of Jilin Province (no: 20210401060YY).

## Conflict of interest

The authors declare that the research was conducted in the absence of any commercial or financial relationships that could be construed as a potential conflict of interest.

## Publisher’s note

All claims expressed in this article are solely those of the authors and do not necessarily represent those of their affiliated organizations, or those of the publisher, the editors and the reviewers. Any product that may be evaluated in this article, or claim that may be made by its manufacturer, is not guaranteed or endorsed by the publisher.

## Supplementary material

The Supplementary material for this article can be found online at: https://www.frontiersin.org/articles/10.3389/fnut.2023.1217862/full#supplementary-material
