# Supplementary material for: Structural characterization and antioxidant activity of pectic polysaccharides from Veronica peregrina L
Source: Front Nutr. 2023 Jun 29;10:1217862. doi: 10.3389/fnut.2023.1217862 (PMC10345500; doi:10.3389/fnut.2023.1217862)
Supplement: Supplementary file 1 [file Data_Sheet_1.docx]

Supplementary Material

**Structural characterization and antioxidant activity of pectic polysaccharides from *Veronica peregrina* L.**

**Su Yan, Xianbin Liu, Yuwen Wang, Xiaomin Yang, Lu Bai, Lin Sun, Yifa Zhou, Sisi Cui***

*** Correspondence:** Sisi Cui: cuiss100@nenu.edu.cn

# Supplementary Figures

**
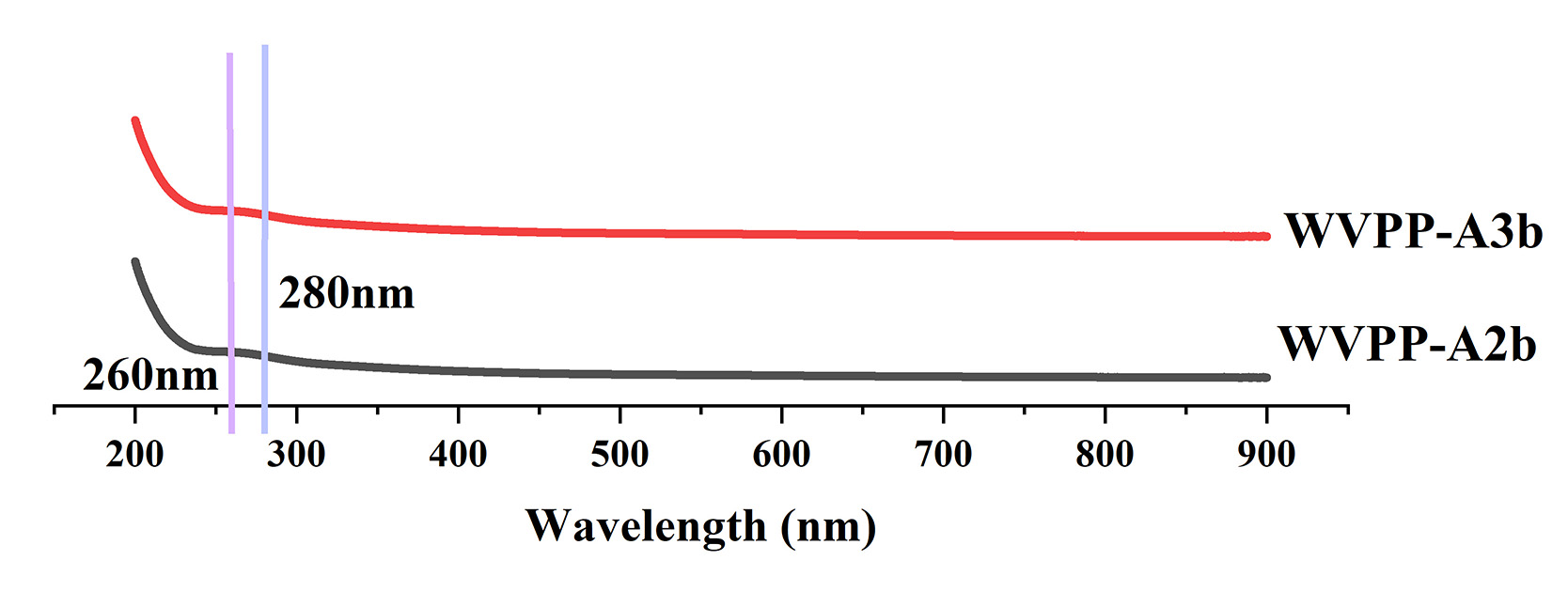
**

**Supplementary Figure 1.** UV analysis of WVPP-A2b and WVPP-A3b.


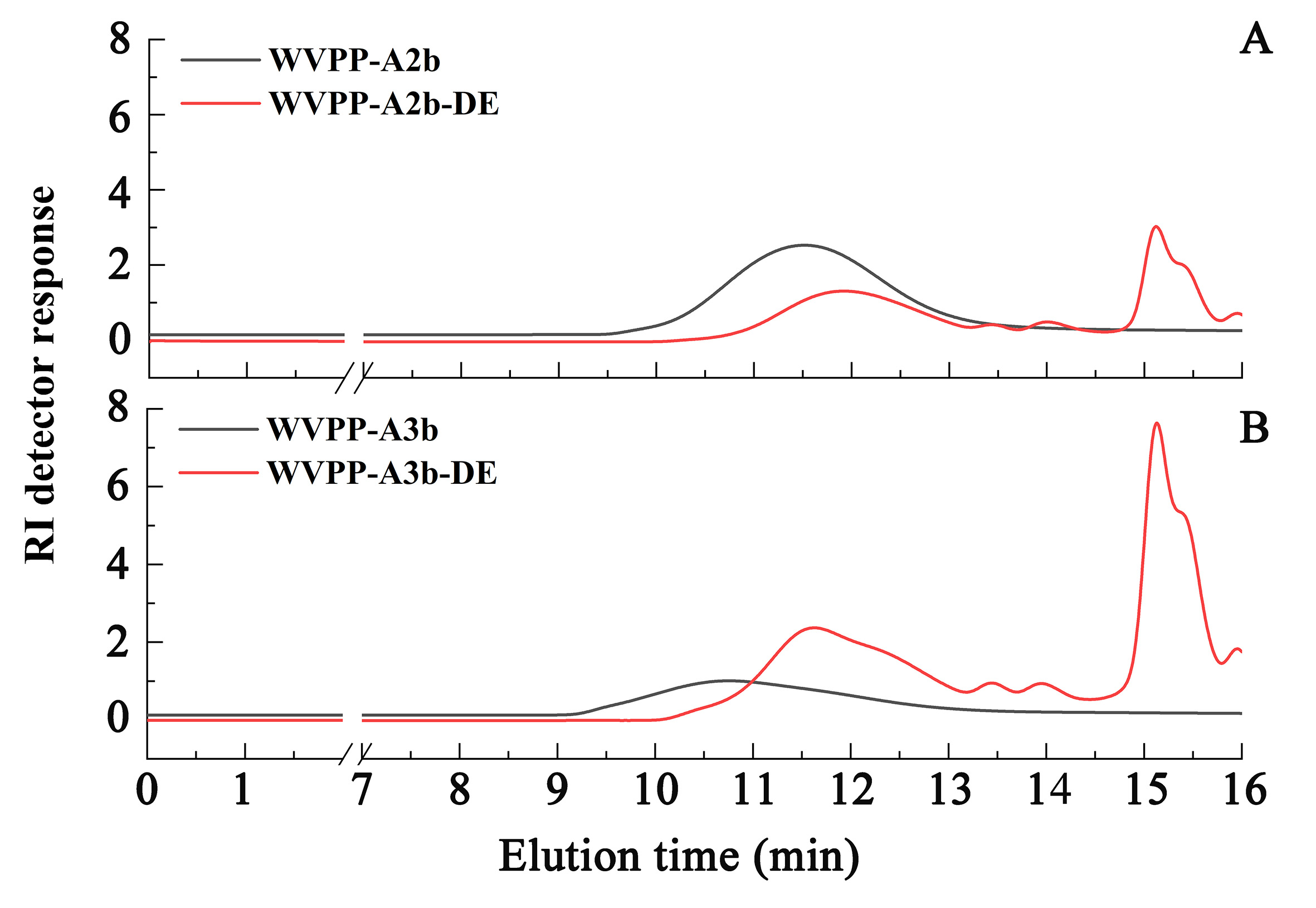


**Supplementary Figure 2.** HPGPC elution profiles of the two pectins and their de-esterified and enzymatic hydrolysates.


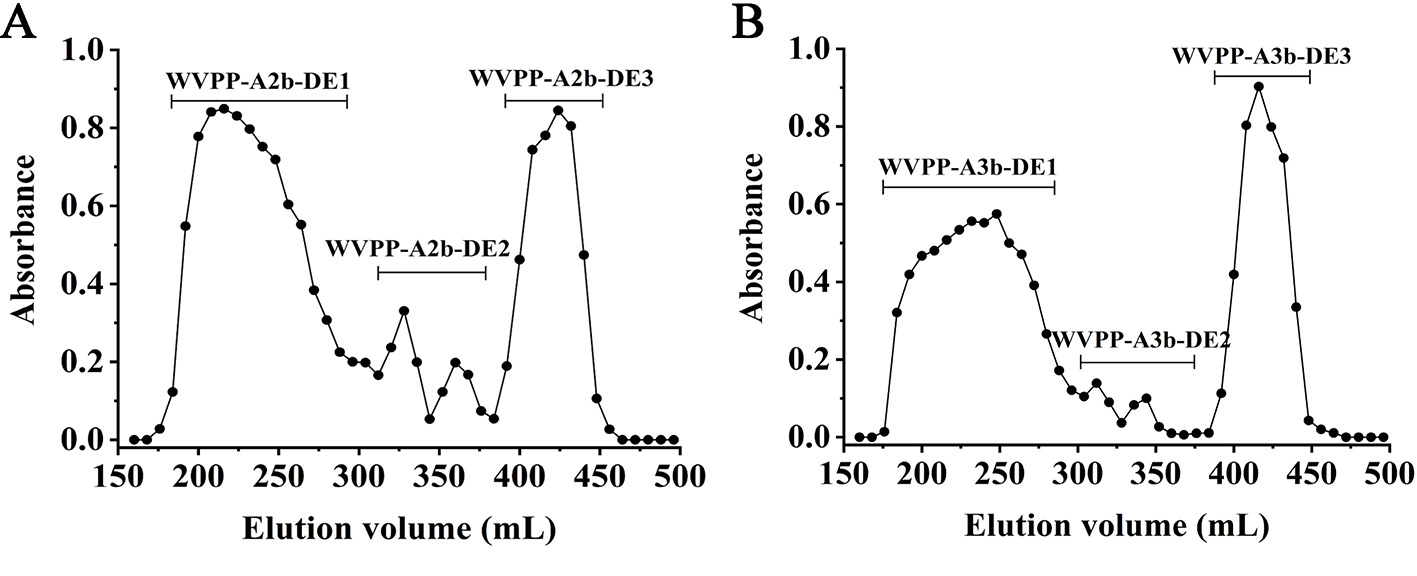


Supplementary Figure 3. The elution profiles of the de-esterified and enzymatic hydrolysates on Sephadex G-75 column (A) WVPP-A2b, (B) WVPP-A3b.
